# Protection of Fecal Microbiota Transplantation in a Mouse Model of Multiple Sclerosis

**DOI:** 10.1155/2020/2058272

**Published:** 2020-08-05

**Authors:** Kanglan Li, Shouchao Wei, Li Hu, Xiaojian Yin, Yingren Mai, Chunmei Jiang, Xiaoping Peng, Xingxing Cao, Zhongkai Huang, Haihong Zhou, Guoda Ma, Zhou Liu, Huiliang Li, Bin Zhao

**Affiliations:** ^1^Guangdong Key Laboratory of Age-Related Cardiac and Cerebral Diseases, Institute of Neurology, Department of Neurology, Affiliated Hospital of Guangdong Medical University, Zhanjiang, Guangdong 524001, China; ^2^Department of Histology and Embryology, Guangdong Medical University, Zhanjiang, China; ^3^Department of Neurosurgery, Affiliated Hospital of Guangdong Medical University, Guangdong Medical University, Zhanjiang, China; ^4^Wolfson Institute for Biomedical Research, University College London, London WC1E 6BT, UK

## Abstract

Given the growing evidence of a link between gut microbiota (GM) dysbiosis and multiple sclerosis (MS), fecal microbiota transplantation (FMT), aimed at rebuilding GM, has been proposed as a new therapeutic approach to MS treatment. To evaluate the viability of FMT for MS treatment and its impact on MS pathology, we tested FMT in mice with experimental autoimmune encephalomyelitis (EAE), a mouse model of MS. We provide evidence that FMT can rectify altered GM to some extent with a therapeutic effect on EAE. We also found that FMT led to reduced activation of microglia and astrocytes and conferred protection on the blood-brain barrier (BBB), myelin, and axons in EAE. Taken together, our data suggest that FMT, as a GM-based therapy, has the potential to be an effective treatment for MS.

## 1. Introduction

Multiple sclerosis (MS) commonly occurs as a progressive central nervous system (CNS) disease, characterized by inflammation, demyelination, and axonal loss in the brain and spinal cord [1]. T cell-mediated inflammatory pathology and genetic factors are closely involved in the development of MS, causing damage to myelin sheaths surrounding neuronal axons and accumulation of neurological deficits [2–4]. Environmental factors also play a driving role in the pathogenesis of MS, such as geographical latitude, vitamin D3 deficiency, early life obesity, passive smoking, Epstein-Barr virus infection, dietary habits (especially high salt and fat diet), stress, and gut microbiota (GM) [5]. Study has shown that transplanting the intestinal microbiota of autism spectrum disorder patients into germ-free mice and that colonization of the microbiota induced typical autism spectrum disorder behaviors [6]. Germ-free mice developed severe MS symptoms after microbiota transplants from MS patients compared with transplanted healthy controls [7]. MS patient-derived microbiota resulted in a spontaneous EAE in a transgenic mouse model [8]. Human bacteria was transferred to mice can be detected and was a shift of the microbiota over time [9]. Notably, accumulating new evidence points to a link between altered intestinal microbiota and MS pathogenesis [10–15].

Investigation of GM has revealed significantly altered abundances of certain bacterial genera in MS patients compared to healthy controls [16]. Moreover, germ-free mice prove to be resistant to experimental autoimmune encephalomyelitis (EAE), a commonly used animal model of MS [17, 18]. Together, these studies imply a causal association between GM and MS. Although the mechanisms underlying the role of GM in MS are still elusive, GM-based therapeutic strategies hold the promise of new treatments for MS.

Fecal microbiota transplantation (FMT) appears to be an effective treatment for *Clostridium difficile* infection and inflammatory bowel syndrome, able to restore GM diversity to some extent [19, 20]. Some case reports suggest that FMT may help improve symptoms of epilepsy and Parkinson's disease [21, 22]. Interestingly, one study found that after FMT treatment for constipation, three wheelchair-bound MS patients had so dramatic improvement in neurological symptoms that they regained the ability to walk unassisted [23]. Therefore, FMT has the potential to be an innovative therapy for MS. Here, we evaluate the effect of FMT on EAE and explore possible mechanisms behind it. Our data reveal that FMT can improve the clinical outcome of EAE by modulating GM, reducing glial inflammatory response and conferring protection on the blood-brain barrier (BBB), myelin, and axons.

## 2. Materials and Methods

### 2.1. Animals

Four- to five-week-old female C57BL/6 mice were purchased from Guangdong Medical Laboratory Animal Center and raised in pathogen-free conditions in an animal facility at Guangdong Medical University (GMU). Mice were allowed 1 week's habituation before being used for experiments. Animal care and all procedures complied with the guidelines of GMU Experimental Animal Ethics Committee and national laws and regulations of China for use of animals in biomedical research.

### 2.2. EAE Induction

EAE induction was based on a published protocol [24]. Murine myelin oligodendrocyte glycoprotein (MOG) 35–55 peptide (MOG35–55, MEVGWYRSPFSRVVHLYRNGK) was synthesized with >99% purity (SciLight Biotechnology, China). C57BL/6 mice were injected with 200 *μ*g MOG35–55 emulsified in 100 *μ*g of complete Freund's adjuvant (CFA, Sigma) and an additional 400 *μ*g *Mycobacterium tuberculosis* H37RA (BD Biosciences) by subcutaneous injection into the flanks. These mice were also given 400 ng pertussis toxin (List Biological Laboratories) in 100 *μ*l phosphate buffered saline (PBS) on the same day and 2 days later by intraperitoneal injection. In addition, MOG35–55 was administered again 1 week later. Clinical scores were recorded daily for 42 days postimmunization. Neurological function was scored on a 0-5 scale: 0, no signs of disease; 1, partial loss of tail tonicity; 2, tail paralysis; 3, ataxia and/or paresis of hind limbs; 4, complete paralysis of hind limbs; and 5, moribund or death [24]. A cumulative clinical score was the sum of all neurological function scores from onset to day 42. Day of onset was when an animal first exhibited neurological signs of disease.

### 2.3. Fecal Microbiota Transplantation (FMT)

C57BL/6 mice (3/cage) were placed in empty autoclaved cages (no bedding) and allowed normal bowel movement. At least twelve fecal pellets were collected from each cage using sterile filter paper, promptly placed in 3 ml sterile PBS, homogenized for 2 min with a glass pestle and spun at 800 rpm for 3 min before collecting the supernatant for transplantation. Immunized mice were randomly divided into two groups: one group (FMT group) was given 200 *μ*l per mouse fresh fecal supernatant via oral gavage daily for 42 consecutive days postimmunization, whereas for the other group (EAE group), fecal supernatant was replaced with sterile saline.

### 2.4. Sample Collection and Microbiota Analysis

Fresh feces were collected and immediately stored at -80°C. Fecal microbiota DNA was recovered with the PowerFecal DNA Kit (Qiagen). The V3-V4 region of 16S rRNA gene was amplified by using a pair of primers: 338F, 5′-ACTCCTACGGGAGGCAGCAG-3′, and 806R, 5′-GGACTACHVGGGTWTCTAAT-3′. PCR amplification products were sequenced by paired-end sequencing (Majorbio, China).

### 2.5. Tissue Preparation

At day 42 postimmunization, mice were perfused transcardially with ice-cold saline under terminal anesthesia. Mice used for immunostaining were further perfused with 4% paraformaldehyde. Dissected brain and thoracic spinal cord tissues were fixed in 4% paraformaldehyde for 12-24 h and then immersed in 20% and 30% sucrose each for 1 day. The tissues were embedded in Tissue-Tek OCT (Sakura), frozen, and cut into 25 *μ*m thick spinal cord cross sections and brain coronal sections. For evaluation of BBB leakage, 4% Evans blue (Sigma) in PBS was injected into the tail vein of mice (3 *μ*l/g) under anesthesia two hours before perfusion.

### 2.6. Immunofluorescence Staining

To detect Claudin 5 expression, thoracic spinal cord sections were sealed with blocking buffer (10% sheep serum albumin and 0.3% Triton X-100 in PBS) for 30 min at 37°C, followed by incubation with anti-Claudin 5 antibody (1 : 200, ab15106, Abcam, USA) at 4°C overnight. For other examinations by immunostaining, brain sections were sealed with anti-Iba1 (1 : 100, ab153696, Abcam, USA), anti-GFAP (1 : 200, Cat.#12389, CST, USA), or anti-MBP (1 : 100, Cat.#13344, CST, USA) together with anti-NF-L antibody (1 : 400, Cat.#AB9568, Millipore, USA) at 4°C for 48 h. Alexa Fluor® 488-conjugated goat anti-rabbit secondary antibody (1 : 800, ab150077, Abcam, USA) was then added on its own or together with Alexa Fluor® 555-conjugated goat anti-mouse antibody (1 : 800, ab150118, Abcam, USA) for incubation at 37°C for 1 h. Nuclei were counterstained with Hoechst 33342 (Cat.#C0030, Solarbio, Beijing, China) for 10 min. Photomicrographs were taken with a confocal microscope (Leica TCS SP5 II).

### 2.7. Transmission Electron Microscopy

Thoracic spinal cord tissues were postfixed in osmium tetroxide and processed for transmission electron microscopy. Electron micrographs were taken with a Jem-1400 transmission electron microscope.

### 2.8. Western Blotting

The protein was extracted from brain tissues using an Ambion PARIS kit (Solarbio, Beijing, China) with addition of phosphatase and protease inhibitor cocktails (Roche). The Pierce BCA Protein Assay Kit (Cat.#23227, Thermo Fisher) was used to detect protein concentrations. The proteins were separated by SDS-PAGE electrophoresis, and a blotted membrane was incubated with blocking buffer and then with anti-Claudin 5 (1 : 500, ab15106, Abcam, USA), anti-Iba1 (1 : 1000, ab153696, Abcam, USA), anti-GFAP (1 : 1000, Cat.#12389, CST, USA), anti-MBP (1 : 1000, Cat.#13344, CST, USA), or anti-NF-L (1 : 1000, Cat.#AB9568, Millipore, USA) primary antibody at 4°C overnight before addition of horseradish peroxidase- (HRP-) linked goat anti-rabbit (1 : 1000, Cat.#7074, CST, USA) or goat anti-mouse secondary antibody (1 : 25000, Cat.E030110-01, EarthOx, USA). Detection of *β*-tubulin was performed on stripped membranes with anti-*β*-tubulin (1 : 1000, ab179513, Abcam, USA) primary antibody to control for protein loading. Protein signal was visualized with the LumiGLO chemiluminescent substrate (Cat.#7003S, CST, USA), and target protein expression was normalized as fold change relative to *β*-tubulin expression using Photoshop for quantitative analysis.

### 2.9. Statistical Analysis

Statistical analysis was performed by the Mann–Whitney *U* test, followed by a linear discriminant analysis for phylum level changes. The SPSS 17.0 software was used for statistical analysis including two-way analysis of variance (ANOVA) followed by a Bonferroni test for comparing clinical scores and unpaired *t*-tests for other comparisons with means and *p* values calculated. Statistical graphs were generated with GraphPad Prism 5. *p* < 0.05 was considered statistically significant.

## 3. Results

### 3.1. FMT Modulates GM in EAE

**Figure 1:**
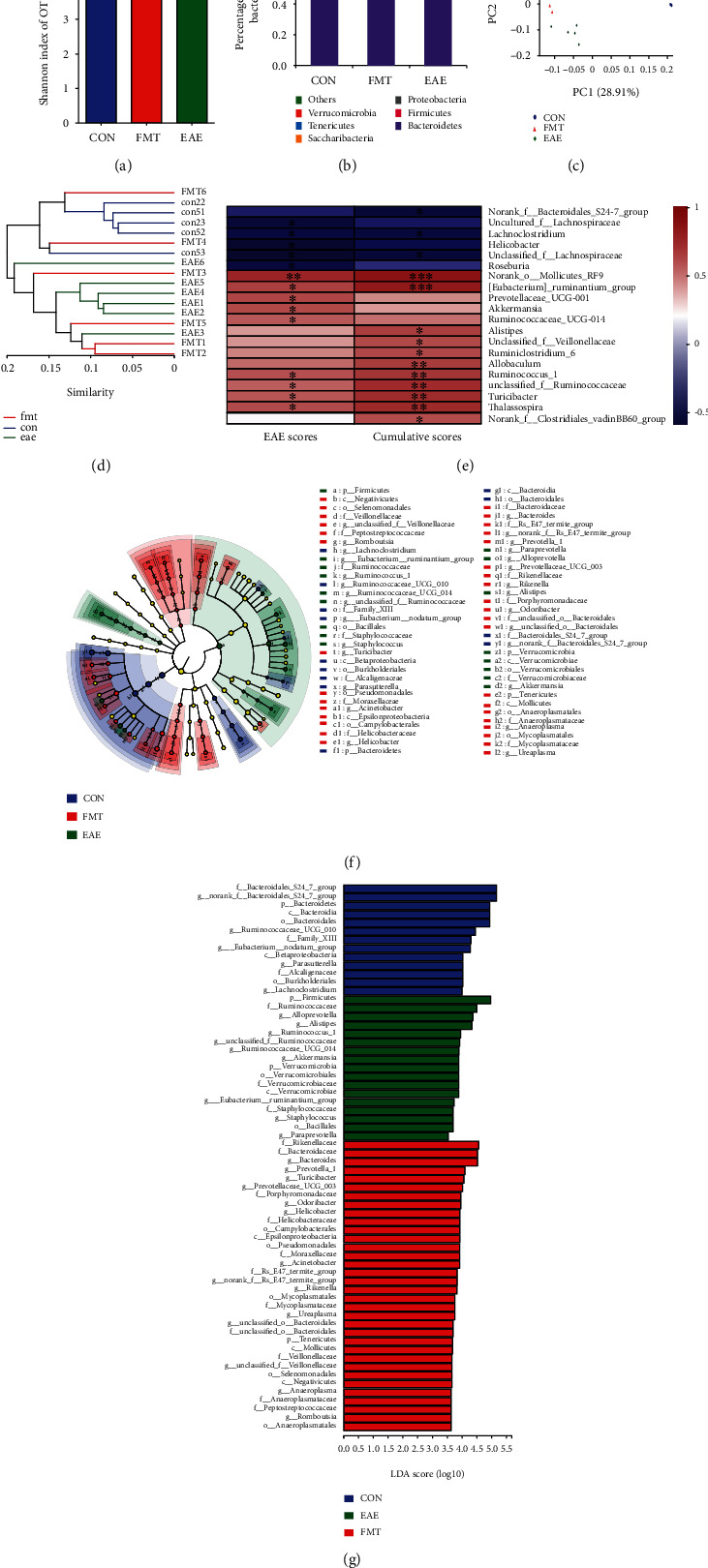
FMT modulates GM in EAE. (a) Chart of the Shannon index values for evaluation of GM *α*-diversity. The Shannon index for GM was significantly increased in EAE mice (*n* = 6) compared to normal controls (CON, *n* = 5); an in-between value was found in FMT-treated immunized mice (FMT; *n* = 6) without statistical significance. (b) Chart of relative abundance of gut bacterial taxa (phylum level) performed with the Mann–Whitney *U* test, corrected by FDR between the two groups. The figure was created by used unfiltered OUT table. (c) PCoA plot (PC1/PC2) of unweighted UniFrac distances illustrating clustering separation between samples from different mouse groups. (d) Hierarchical clustering tree on OTU level (weighted UniFrac). (e) The correlation heatmap chart performed with a Spearman's correlation of bacterial abundance with EAE scores and cumulative scores. The *R* value is shown in different colors in the figure. The legend on the right is the color interval of different *R* values (∗0.01 < *p* < 0.05 and ∗∗0.001 < *p* < 0.01). (f) LEfSe cladogram showing differentially abundant gut bacterial taxa. The diameter of each dot is proportional to its effect size. Each ring (from inside to outside) represents a taxonomic level from kingdom to genus, the cladogram was made on filtered data, and only taxa with greater than 0.1% relative abundance. (g) LDA scores of abundant gut bacterial taxa (LDA score threshold >2.0). k: kingdom; p: phylum; c: class; o: order; f: family; g: genus.

To examine the effect of FMT on GM structure in EAE, fecal samples were collected from EAE mice (*n* = 6), FMT-treated immunized mice (*n* = 6), and normal controls (*n* = 5) 42 days postimmunization for DNA extraction and 16S rRNA gene sequencing. Acquired sequencing data were analyzed for assessment of GM diversity. We first evaluated GM *α*-diversity with the Shannon index, which takes account of both the richness and evenness of a microbial community. The Shannon index was significantly increased for GM in EAE mice compared to normal controls (4.38 ± 0.15 vs. 3.89 ± 0.26, *p* = 0.008113), indicating altered GM diversity in EAE; an in-between value was discovered in FMT-treated EAE mice without statistical significance (Figure 1(a)), suggesting that FMT attenuated the increase in the Shannon index caused by the development of EAE. Therefore, these results hint that FMT is conducive to restoring altered intestinal microbiota diversity in EAE. We next evaluated GM *β*-diversity, which accounts for the dissimilarity between different microbial communities. Principal coordinates analysis (PCoA) of unweighted UniFrac distances showed clear clustering separation between samples from these three mouse groups on scores plot for principal component 1 (PC1, 28.91%) and PC2 (13.45%), illustrating differing GM diversity between these mouse groups (Figure 1(c)). The *β*-diversity distance matrix was presented for hierarchical clustering analysis to calculate the phylogenetic evolutionary relationships of each species and the distance between samples; pairwise intergroup UniFrac distances further quantitatively detected the variation occurring on different lineages among samples. Analysis showed that FMT was closer to the control than the EAE mice on the OTU level (Figure 1(d)).

Compared with EAE mice, intestinal bacterial phyla *Bacteroidetes* (*p* = 0.0081) were more abundant in normal controls, whereas *Firmicutes* (*p* = 0.0081), *Tenericutes* (*p* = 0.0081), and *Cyanobacteria* (*p* = 0.0354) were less abundant. FMT-treated EAE mice presented changed abundances of *Verrucomicrobia* (*p* = 0.0091), four intestinal bacterial phyla *Bacteroidetes*, *Firmicutes*, *Tenericutes*, and *Cyanobacteria*, all of which that shifted towards the levels observed in normal controls (Figure 1(b)) even if it is not statistically significant. Those suggest that FMT can to a certain degree remedy altered GM structure in EAE.

To determine which bacteria was associated with the severity of the neurological function score, we performed a Spearman's correlation of bacterial abundance with EAE scores and cumulative disease scores (Figure 1(e)). Both *Lachnoclostridium* and *Unclassified_f_Lachnospiraceae* showed negative correlation in EAE scores and EAE cumulative scores. Five kinds of genus including *norank_o_Mollicutes_RF9*, *[Eubacterium]_ruminantium_group*, *unclassified_f_Ruminococcaceae*, *Turicibacter*, *Ruminococcus_1*, and *Thalassospira*were positively correlated with EAE scores and EAE cumulative scores. However, *uncultured_f_Lachnospiraceae*, *Helicobacter*, *Roseburia*, and *norank_f_Bacteroidales_S24-7_group* were found to be negatively correlated with EAE scores and cumulative scores, respectively. *Prevotellaceae_UCG-001*, *Akkermansia*, and *Ruminococcaceae_UCG-014* showed positive correlation in EAE score, as well as *Alistipes*, *unclassified_f_Veillonellaceae*, *Ruminiclostridium_6*, *Allobaculum*, and *norank_f_Clostridiales_vadinBB60_group* in cumulative scores. These results indicated that different genus of bacteria contributes differently to EAE neurological function score.

Analysis of the GM profiles of these 3 mouse groups using linear discriminant analysis (LDA) of effect size (LEfSe) identified differentially abundant bacterial taxa (LDA score threshold > 2.0), which reflected the effect of EAE and FMT treatment on the abundances of gut bacterial taxa. EAE caused a marked decrease in the abundances of 13 gut bacterial taxa (*g_norank_f_Bacteroidales_S24_7_group*, *f_Bacteroidales_S24_7_group*, *p_Bacteroidetes*, *c_Bacteroidia*, *o_Bacteroidales*, *g_Ruminococcaceae_UCG_010*, *f_Family_XIII*, *g_Eubacerium_nodatum_group*, *c_Betaproteobateria*, *g_Parasutterella*, *f_Alcaligenaceae*, *o_Burkholderiales*, and *g_Lachnoclostridium*), made up of 1 phylum, 2 classes, 2 orders, 3 families, and 5 genera. FMT treatment for EAE decreased the abundances of 17 bacterial taxa (*p_Fimicutes*, *f_Ruminococcaceae*, *g_Alloprevotella*, *g_Alistipes*, *g_Ruminococcus_1*, *g_unclassified_f_Ruminococcaceae*, *g_Ruminococcaceae_UCG_014*, *g_Akkermansia*, *p_Verrucomicrobia*, *o_Verrucomicrobiales*, *f_Verrucomicrobiaceae*, *c_Verrucomicrobiae*, *g_Eubacterium_ruminantium_group*, *f_Staphylococcaceae*, *g_Staphylococcus*, *o_Bacillales*, and *g_Paraprevotella*) consisting of 2 phyla, 1 class, 2 orders, 3 families, and 9 genera (including *Akkermansia*) and increased the abundances of 34 bacterial taxa (*f_Rikenellaceae*, *f_Bacteroidaceae*, *g_Bacteroides*, *g_Prevotella_1*, *g_Turicibacter*, *g_Prevotellaceae_UCG_003*, *f_Porphyromonadaceae*, *g_Odoribacter*, *g_Helicobacter*, *f_Helicobacteraceae*, *o_Campylobacterales*, *c_Epsilonproteobacteria*, *o_Pseudomonadales*, *f_Moraxellaceae*, *g_Acinetobacter*, *f_Rs_E47_termite_group*, *g_norank_f_Rs_E47_termite_group*, *g_Rikenella*, *o_Mycoplasmatales*, *f_Mycoplasmataceae*, *g_Ureaplasma*, *g_unclassified_o_Bacteroidales*, *f_unclassified_o_Bacteroidales*, *p_Tenericutes*, *c_Mollicutes*, *f_Veillonellaceae*, *g_unclassified_f_Veillonellaceae*, *o_Selenomonadales*, *c_Negativicutes*, *g_Anaeroplasma*, *f_Anaeroplasmataceae*, *f_Peptostreptococcaceae*, *g_Romboutsia*, and *o_Anaeroplasmatales* increased in FMT mice) comprising 1 phylum, 3 classes, 5 orders, 11 families, and 14 genera (including *Prevotella*) in GM (Figures 1(f) and 1(g)). Taken together, these results demonstrate that FMT can modulate GM, thereby to some extent rectifying altered GM composition in EAE.

### 3.2. FMT Has a Therapeutic Effect on EAE

**Figure 2:**
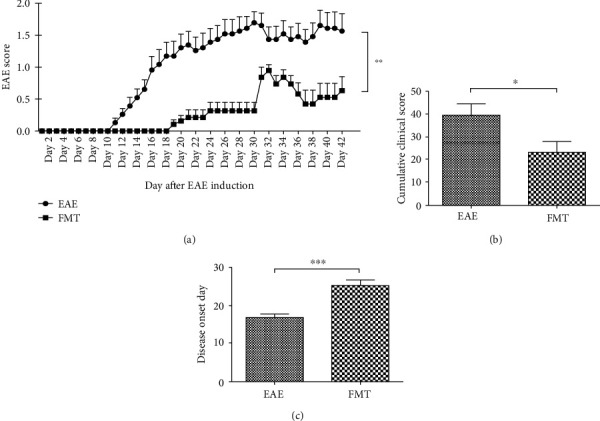
FMT has a therapeutic effect on EAE. (a) Chart of clinical scores for EAE controls and FMT-treated immunized mice after EAE induction. FMT led to decreased clinical scores through the clinical course of EAE (mean ± SEM; *n* = 10/group; ∗∗*p* < 0.01). (b, c) Charts of cumulative clinical scores (b) and disease onset days (c) after EAE induction indicating reduced clinical severity and delayed disease onset after FMT treatment (mean ± SEM; *n* = 10/group; ∗*p* < 0.05 and ∗∗∗*p* < 0.001).

Study of human patients has revealed distinct GM in MS [25], and research with animal models of MS has discovered that modulating GM with antibiotics and probiotics can decrease EAE clinical severity [26]. To find out the effect of FMT on EAE clinical symptoms, we evaluated clinical scores of immunized mice with versus without FMT treatment. FMT-treated mice (*n* = 10) displayed alleviated clinical symptoms evidenced by significantly reduced clinical scores (*p* < 0.0001) and cumulative disease scores (*p* < 0.05) compared with EAE controls (*n* = 10) through the clinic course of EAE (Figures 2(a) and 2(b)). Furthermore, FMT treatment resulted in a delay in the onset of EAE (*p* < 0.0001, Figure 2(c)). Therefore, FMT with fecal matter from normal donors proved effective in slowing down EAE development and relieving EAE symptoms.

### 3.3. FMT Prevents BBB Leakage in EAE

**Figure 3:**
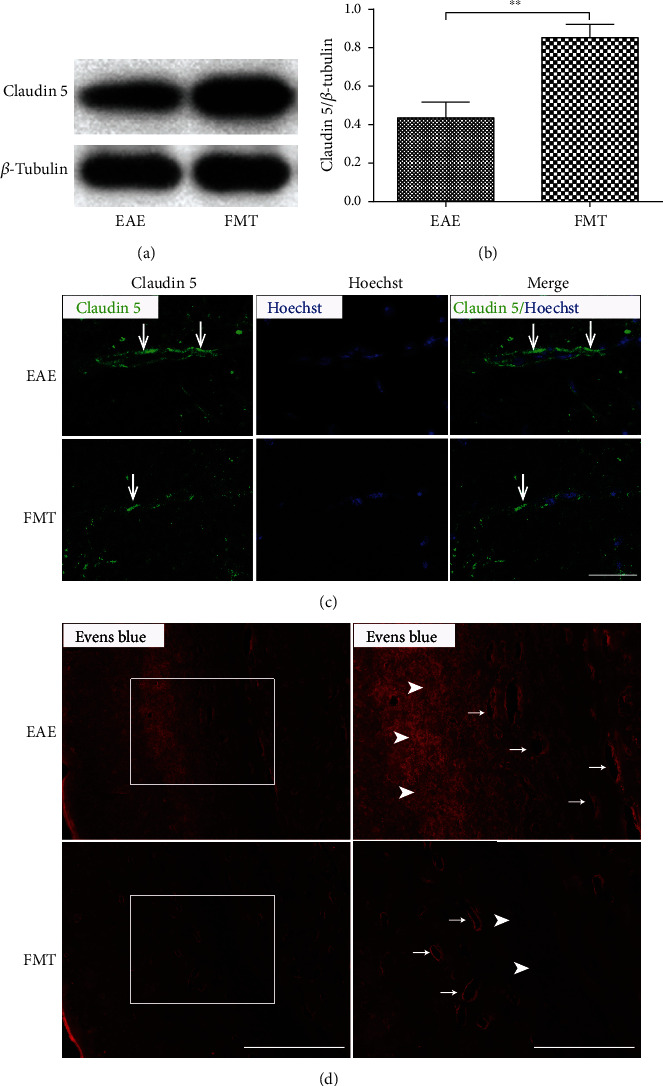
FMT prevents BBB leakage in EAE. (a) Claudin 5 expression in thoracic spinal cord tissues collected from EAE controls and FMT-treated EAE mice revealed by Western blot with *β*-tubulin as loading control. (b) Chart of quantified Western blot results showing increased levels of Claudin 5 expression (normalized by *β*-tubulin) after FMT treatment (mean ± SEM; *n* = 6/group; ∗∗*p* < 0.01). (c) Representative immunostaining images of Claudin 5 expression (green) in spinal cord sections. The nucleus was stained with Hoechst (blue). Scale bar: 100 *μ*m. (d) Representative images of Evans blue dye extravasation (red) in the subcortical white matter of brain showing the presence of dye in both blood vessels and brain parenchyma in EAE controls (upper panels) in contrast to appreciably reduced dye presence in brain parenchyma in FMT-treated EAE mice (lower panels). Each right panel shows a high magnification image of the area inside the white box (left). Scale bars: 400 *μ*m (left) and 200 *μ*m (right). Representative brain parenchyma and blood vessels are indicated by arrowheads and arrows, respectively.

The BBB is compromised during the development of MS and EAE, allowing immune cells to infiltrate CNS and attack myelin [27]. Because GM is thought able to regulate BBB permeability [28–30], we then investigated whether FMT can help prevent BBB impairment in EAE. By Western blotting, we found that the expression of Claudin 5, a tight junction protein responsible for BBB barrier function [31], was dramatically increased in brain tissue in FMT-treated immunized mice compared to EAE controls (*n* = 6) (Figures 3(a) and 3(b)), which was further verified by immunostaining of brain sections (*n* = 3) (Figure 3(c)). In addition, Evans blue dye staining showed an appreciable reduction in dye presence in brain parenchyma after FMT treatment (*n* = 1) (Figure 3(d)). Together, these results prove that FMT treatment can lead to improved BBB integrity in EAE, preventing BBB leakage.

### 3.4. FMT Confers Protection on Myelin and Axons in EAE

**Figure 4:**
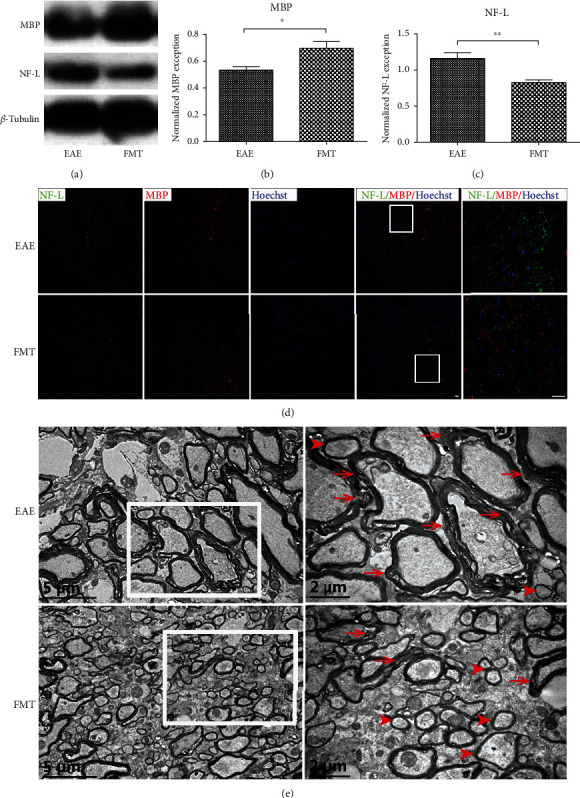
FMT confers protection on myelin and axons in EAE. (a) MBP and NF-L protein expression in the brain of EAE controls and FMT-treated EAE mice shown by Western blot with *β*-tubulin as loading control. (b, c) Charts of quantified Western blot results showing increased levels of MBP (b) and decreased levels of NF-L (c) expression (normalized by *β*-tubulin) after FMT treatment (mean ± SEM; *n* = 6/group; ∗*p* < 0.05 and ∗∗*p* < 0.01). (d) Immunofluorescence imaging of MBP (green) and NF-L (red) expression in the corpus callosum of the mouse brain. Nuclear staining was by Hoechst (blue). Each rightmost panel shows a high magnification image of the area inside the white box. Scale bar: 25 *μ*m. (e) Transmission electron microscopy (TEM) imaging of myelin sheaths in the thoracic spinal cord. Each high magnification image (right) shows the area enclosed by the white box (left). Representative normal and damaged myelin sheaths are indicated by arrowheads and arrows, respectively.

To assess the influence of FMT on myelin and axons, we examined the expression of myelin basic protein (MBP), which is expressed in myelin, and neurofilament light chain protein (NF-L), whose release reflects axonal damage, in brain tissue. Significantly increased MBP expression and decreased NF-L expression were detected in brain tissue in FMT-treated EAE mice compared to saline-treated controls by Western blotting (Figures 4(a)–4(c)) and by immunostaining of brain sections as well (Figure 4(d)), indicating an increase in the number of normal myelin sheaths and a decrease in myelin disintegration and axon damage after FMT treatment. Moreover, transmission electron microscopy (TEM) verified lessened demyelination and increased the presence of intact myelin sheaths in the thoracic spinal cord after FMT treatment (Figure 4(e)). All together, these data point to a protective effect conferred by FMT on myelin and axons in EAE.

### 3.5. FMT Alleviates Microglia and Astrocyte Activation in EAE

**Figure 5:**
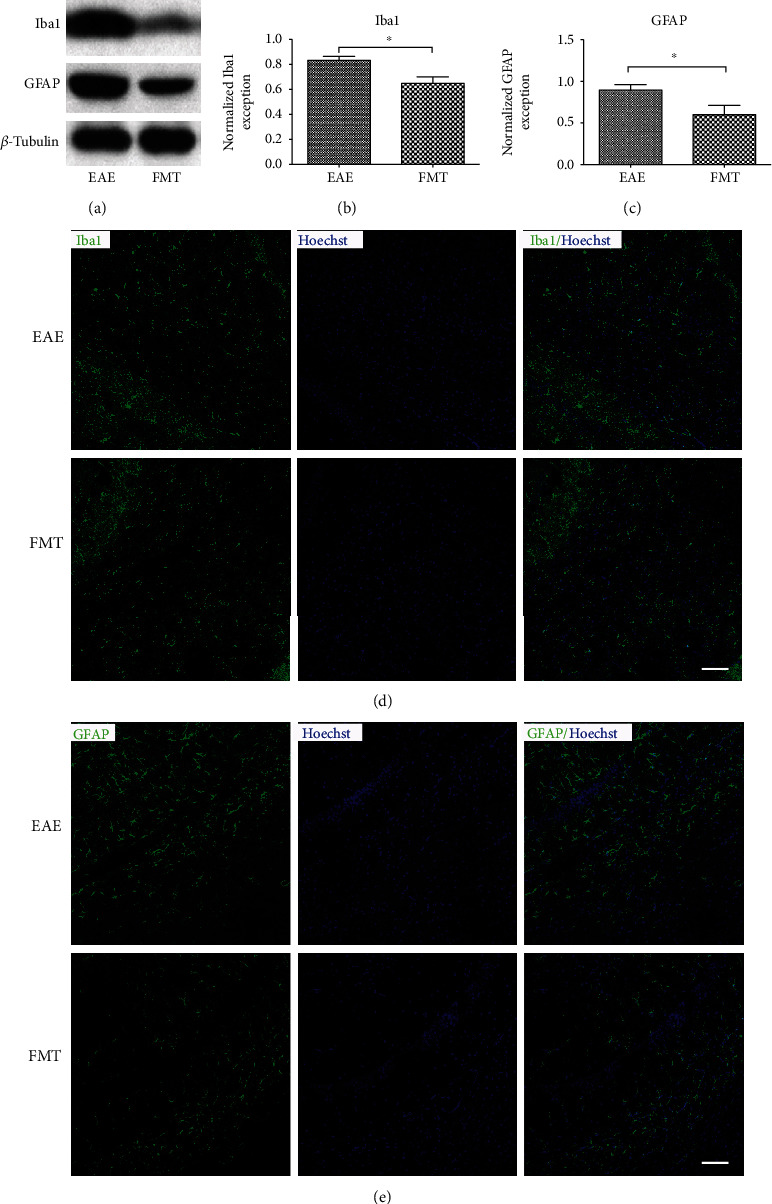
FMT alleviates immune cells and astrocyte activation in EAE. (a). Expression of Iba1 and GFAP protein in the brain of EAE controls and FMT-treated EAE mice shown by Western blot with *β*-tubulin as loading control. (b, c). Charts of quantified Western blot results indicating reduced levels of Iba1(b) and GFAP (c) expression (normalized by *β*-tubulin) after FMT treatment (mean ± SEM; *n* = 6/group; ∗*p* < 0.05). (d, e) Immunofluorescence imaging of cells expressing Iba1 (green; d) and GFAP (green; e) in brain sections with the nucleus stained by Hoechst (blue). Scale bar: 100 *μ*m.

Microglia and astrocytes are known to contribute to the inflammatory pathology of MS [32], and some studies hint at a connection between their activation and GM composition [33, 34]. To find out the impact of FMT on microglia and astrocyte activation, we examined the expression of ionizing calcium-binding adaptor molecule 1 (Iba1, immune cells marker, which is not specific for microglia and infiltrating monocyte) and glial fibrillary acidic protein (GFAP, astrocyte marker) with Western blotting and discovered that the expression of both markers was significantly downregulated in FMT-treated EAE mice compared to saline-treated controls (Figures 5(a)–5(c)). Furthermore, decreased numbers of microglia and astrocytes were observed by immunostaining of brain sections after FMT treatment (Figures 5(d) and 5(e)). Taken together, these data imply subdued glial inflammatory response after FMT treatment in EAE.

## 4. Discussion

An increasing body of evidence reveals GM dysbiosis in MS [16, 25, 35, 36], and rebuilding GM has been proposed as an innovative approach to MS treatment. FMT appears to be the most direct way to reconstruct GM [37, 38] and is in fact an ancient treatment dating back to 1700 years ago [39]. However, whether GM can be restored back to normal by FMT remains unclear. Our data reveal a tendency for GM structure to change towards normal after FMT treatment in EAE with beneficial consequences (Figure 1).

We also found that FMT treatment for EAE markedly reduced the abundance of *Akkermansia* genus (in phylum *Verrucomicrobia*) and elevated the abundance of *Prevotella* genus (in phylum *Bacteroidetes*) in GM (Figures 1(b), 1(f), and 1(g)), which recalls the findings of decreased gut *Akkermansia* after probiotic intervention [40, 41], and increased gut *Prevotella* after disease-modifying treatment [16] and intermittent fasting in MS patients [42]. *Bacteroidetes* is one of the most abundant bacterial phyla inhabiting human gut, and the *Prevotella* genus is a dominant member of this phylum. *Bacteroidetes* ferments dietary fibers to produce short chain fatty acids (SCFAs), which take part in various physiological processes, affecting host health [43–45]. SCFAs have been found to protect the BBB from oxidative stress via nuclear factor, erythroid 2-like 2 (NFE2L2) signaling [46] and exert anti-inflammatory and neuroprotective functions [47–49]. Other favorable effects of SCFAs include attenuating myelin injury by increasing brain acetyl-CoA metabolism [50] and relieving clinical symptoms in EAE mice [49, 50]. A zwitterionic capsular polysaccharide A (PSA) produced by *Bacteroides fragilis* suppresses neuroinflammation by regulating migratory capacity of CD39^+^ CD4 T cell subsets, thus ameliorates EAE [51]. *Bacteroides fragilis* PSA+ regulated CNS demyelination by the induction of highly potent IL-10-producing Treg cells in EAE [52]. In addition, increased gut *Akkermansia* is associated with MS [16, 40], and probiotic treatment for MS patients resulted in decreased *Akkermansia* in GM accompanied by an anti-inflammatory peripheral response [40]. Moreover, increase of gut *Akkermansia* has been implicated in Parkinson's disease [53, 54] and advanced dementia [55]; these results showed a negative role for intestinal *Akkermansia* in CNS disorders. While *Akkermansia* is consistently elevated in MS subjects, however, it may be a compensatory change for a study has shown that transferring *Akkermansia* to mice at EAE onset can ameliorate disease [56].

So far, FMT has been tested in treating a variety of conditions including *Clostridium difficile* infection [42, 57], active ulcerative colitis [58], high-fat diet-induced steatohepatitis [59], metabolic syndrome [60], and CNS diseases such as epilepsy [21] and autism spectrum disorder [61]. Notably, applying FMT to several MS patients [62] achieved promising improvement of clinical outcome and FMT treatment for EAE mice with fecal matter from immunized mice on intermittent fasting ameliorated EAE clinical course [42]. In this study, FMT had a therapeutic effect on EAE, reducing clinical severity and delaying the onset of disease (Figure 2). Hence, our data add more weight to the idea of using FMT as a GM-based treatment for MS.

Currently, how FMT exerts influence on MS remains unclear. Evidence from the EAE model of MS suggests a critical role for GM and its metabolites in the mechanisms behind neuroinflammation and demyelination [17, 18]. Our attempt to rectify altered GM in EAE by FMT led to alleviated neuroinflammation and reduced BBB leakage and damage to myelin and axons (Figures 3–5). These results lend support to GM's involvement in the pathogenesis of EAE and echo previous observations of GM regulating BBB integrity [28], myelination [63], and microglia activation [29, 64]. It is worth noting that two earlier studies experimented with applying FMT to EAE animals, but the fecal matter used for transplantation came from donor animals under specific experimental conditions [42, 65]. FMT from immunized donor mice on intermittent fasting resulted in reduced inflammation and demyelination in EAE recipient mice, proving that GM was part of the reason for fasting to take effect in treating EAE [42]. FMT from Albino Oxford (AO) donor rats, which are highly resistant to EAE induction, to EAE-prone Dark Agouti (DA) recipient rats from birth led to ameliorated EAE symptoms and decreased production of interleukin- (IL-) 17, a proinflammatory cytokine, in the spinal cord [65], hinting that GM can confer resistance to EAE.

Our study has limitations. Firstly, we started FMT treatment before the onset of neurological signs, which is a disadvantage from a translational point of view as treatment for MS is sought after the onset of disease. We also did not analyze the ingredients of the fecal matter used for transplantation.

## 5. Conclusions

In summary, our data demonstrate beneficial effects of FMT in the EAE mouse model of MS, including improved GM composition, ameliorated clinical course, subdued glial inflammatory response, and protection conferred on the BBB, myelin, and axons. Our findings suggest a causal linkage between GM and MS pathogenesis, and therefore, GM has the potential to be a new target of innovative therapies for MS. Further work is needed to unravel the mechanisms underlying the impact of FMT on gut-brain axis and formulate an ideal microbial recipe for MS treatment.

## Data Availability

The raw sequences were deposited in the NCBI Sequence Read Archive (SRA) with the accession number SRP201403.
